# P-395. Clinical and Demographic Characteristics of Community Associated (CA) *Clostridioides difficile*

**DOI:** 10.1093/ofid/ofae631.596

**Published:** 2025-01-29

**Authors:** Adam J Hawco, christopher J Myers, Runda Dahhan, Christine Hurley, Ghinwa Dumyati

**Affiliations:** NY Emerging Infections Program, URMC, West Seneca, New York; University of Rochester, Rochester, New York; University of Rochester, Rochester, New York; University of Rochester, Center for Community Health and Prevention, Rochester, New York; New York Emerging Infections Program and University of Rochester Medical Center, Rochester, New York

## Abstract

**Background:**

The incidence of community associated *Clostridiodes difficile* infections CDI (CA CDI) increased over the last decade; with antibiotic use recognized as a risk factor for infection. In 2017, we noticed an increase in the number of CA cases occurring without recent antibiotic exposure. Our aim was to characterize CA CDI cases both with and without prior antibiotic use to better understand this emerging trend.

**Figure:**
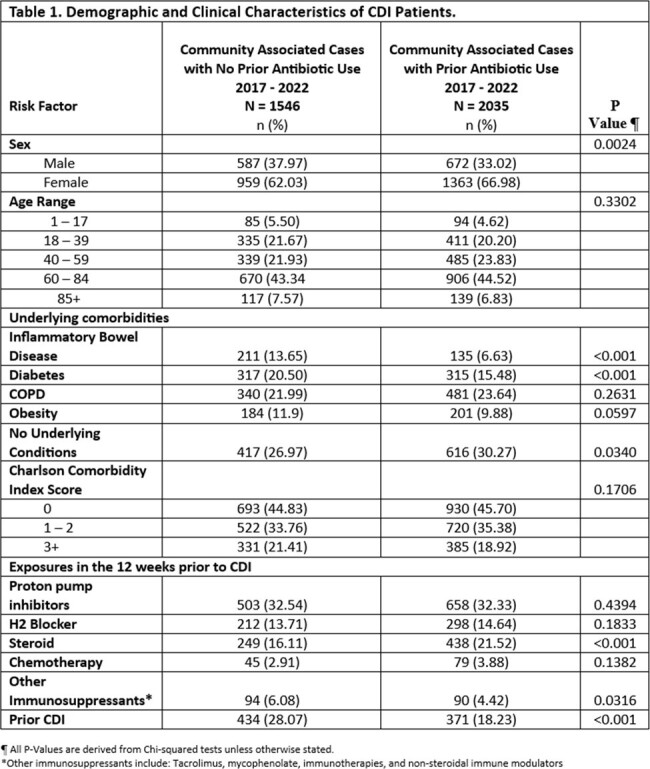

**Methods:**

Active laboratory and population-based surveillance for CDI was performed in Monroe County, NY as part of the CDC Emerging Infections Program between 2017-2022. An incident CA CDI case was defined as a patient with a positive *C. difficile* assay with no prior positive assay in the previous 8 weeks and no evidence of an overnight stay in a healthcare facility in the preceding 12 weeks. Clinical, demographic and exposure data were collected through medical record review. Descriptive and bivariate analyses were then performed.

**Figure:**
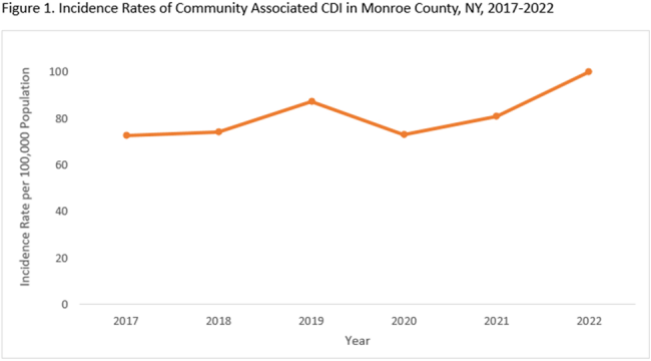

**Results:**

The incidence of CA CDI increased from 72.6 in 2017 to 100/100,000 population in 2022 (Figure 1). Among CA cases that received antibiotics in the 12 weeks prior to their infection, the proportion receiving fluoroquinolones and clindamycin decreased over time (Figure 2). During this period, the proportion of CA CDI cases that did not receive any prior antibiotics increased from 33.7% to 46.9% (Figure 2). Compared to cases with prior antibiotics, cases without prior antibiotic exposure were more likely to be male, on immunosuppressants, have a higher Charlson comorbidity score, have underlying inflammatory bowel disease and prior CDI (Table 1). Prior proton pump inhibitor or H2 blocker use was not significantly different between the two groups.

**Figure:**
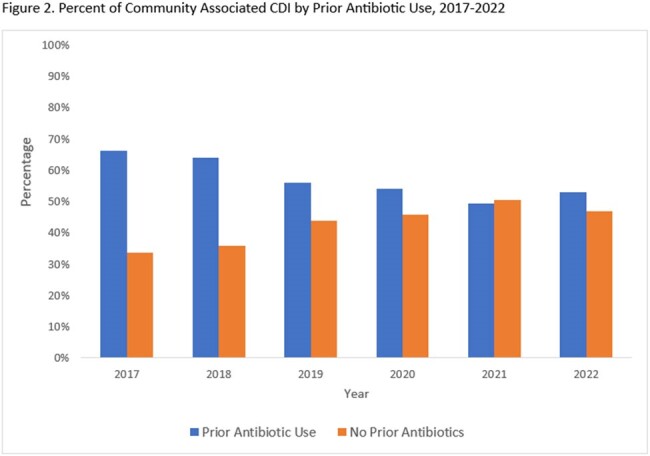

**Conclusion:**

The study observed an increase in the incidence of CA CDI over the study period, with a growing proportion of cases occurring without recent antibiotic exposure. Additionally, the use of high-risk antibiotics decreased over time. These findings suggest that other factors, beyond antibiotic use, may play a significant role in the development of CA CDI. Further research into these emerging risk factors is necessary to inform targeted prevention strategies and address the rising incidence of CA CDI.

**Disclosures:**

**All Authors**: No reported disclosures

